# Unusual features of lattice dynamics in lawsonite near its phase transitions

**DOI:** 10.1038/s41598-022-09890-7

**Published:** 2022-04-13

**Authors:** Filip Kadlec, Dmitry Nuzhnyy, Christelle Kadlec, Jan Petzelt, Maxim Savinov, Stanislav Kamba

**Affiliations:** Institute of Physics, Czech Academy of Sciences, Na Slovance 2, 182 21 Prague 8, Czech Republic

**Keywords:** Ferroelectrics and multiferroics, Phase transitions and critical phenomena

## Abstract

Lattice dynamics of a single crystal of lawsonite were studied over a broad range of frequencies (1 Hz to 20 THz) using impedance, THz time-domain and infrared spectroscopies. Based on polarized spectra of complex permittivity $\hat{\varepsilon }$ measured as a function of temperature between 10 K and 500 K, we analyzed the properties of the two known phase transitions—an antiferrodistortive one near $T_{\mathrm{c}1}=270\,\mathrm{K}$ and a ferroelectric one, occurring at $T_{\mathrm{c}2}=124\,\mathrm{K}$. The former one is accompanied by a flat maximum in the THz-range permittivity $\hat{\varepsilon }_{\mathrm{c}}$ near $T_{\mathrm{c}1}$, which is due to an overdamped polar excitation in the $\mathbf {E} \parallel c$ spectra reflecting the dynamics of water and hydroxyl groups. The strength of this mode decreases on cooling below $T_{\mathrm{c}1}$, and the mode vanishes below $T_{\mathrm{c}2}$ due to hydrogen ordering. At the pseudoproper ferroelectric phase transition, two independent anomalies in permittivity were observed. First, $\hat{\varepsilon }_a$ exhibits a peak at $T_{\mathrm{c}2}=124\,\mathrm{K}$ due to critical slowing down of a relaxation in the GHz range. Second, infrared and THz spectra revealed an optical phonon softening towards $T_{\mathrm{c}2}$ which causes a smaller but pronounced maximum in $\hat{\varepsilon }_b$. Such anomaly, consisting in a soft mode polarized perpendicularly to the ferroelectric axis, is unusual in ferroelectrics.

## Introduction

Lawsonite, $\hbox {CaAl}_2\hbox {Si}_2\hbox {O}_7(\hbox {OH})_2\cdot \hbox {H}_2\hbox {O}$, is a mineral which is abundant in the Earth’s crust and mantle^1^, and it is attracting a particular interest of two fields of study—deep-crust geology and solid state physics. In the former domain, it is usually found in metamorphic rocks^2^, and it is examined as a significant hydrous mineral formed by means of water-rock interactions. As a material playing a role in the vertical transport of elements and water, it is known mainly from areas along subduction zones, however, it occurs supposedly^3^ at depths of up to 250 km, in an environment characterized by high pressures and temperatures. Indeed, lawsonite is stable at these conditions, and it might be even the dominant hydrous material^4^ at pressures between 2.5 and 14 GPa. In its phase diagram, the phase stability zone extends up to temperatures between 740 and $1070\,^{\circ }\hbox {C}$ and pressures of 10–14 GPa. When these limit conditions are exceeded, lawsonite breaks down to other minerals while releasing water vapor^4^. This reaction has been proposed as a possible driving force for intermediate-depth earthquakes occurring in the subduction zones^5,6^. For these reasons, it is important to study the chemical and physical properties of lawsonite. However, despite the presumed abundance at deeper levels, its crystals are rather rarely preserved in the rocks exhumed to the Earth surface^7,8^.

From the point of view of solid-state physics, which we adopt in the following, lawsonite shows a combination of several features rarely occurring simultaneously. It is a natural ferroelectric containing inherently crystal water; single $\hbox {H}_2\hbox {O}$ molecules are stoichiometrically contained in every unit cell. Owing to the simultaneous presence of hydroxyl groups, the protons exhibit, at high temperatures, a positional disorder, and they partially order upon cooling as the crystal undergoes a ferroelectric phase transition. Therefore, lawsonite is also of interest as a solid where mutual dipole–dipole interactions among the water molecules can be studied. In general, their dipole field extends to a multiple of the molecule size. In principle, this is a prerequisite for dipole moment ordering; however, despite the water ubiquity, examples of local ordering of water molecules are extremely rare, because in the vast majority of the known condensed phases, as a rule, the dipole–dipole interactions are strongly counteracted by the short-range hydrogen bonding. A long-standing search for ferroelectric ice^9–11^ has provided no conclusive evidence yet. In contrast, water molecules embedded individually in specific crystals represent quite a different situation; because of a spacing among the closest molecules, amounting typically to a few nanometers, they can not form hydrogen bonds together. Thus, the dipole–dipole interaction may be strong enough to play a key role in molecule ordering. Recently, studies of dielectric properties of water-containing crystals have brought new evidence of low-temperature ordering of water molecules. Specifically, in beryl containing water in nanosized cavities, broadband dielectric spectroscopy of hydrated and anhydrous crystals has shown, at temperatures below $\approx 10\,\mathrm{K}$, a diverging permittivity due to collective vibrations of the water molecules. The frequency of this vibrational mode decreases (softens) with temperature, obeying the Curie-Weiss law^12^. This behavior is generally linked to the appearance of ferroelectricity; however, as its value of Curie temperature $T_{\mathrm{C}}=-20$ K is negative, hydrated beryl ranks among incipient ferroelectrics where the dipole ordering cannot be realized. Interestingly, more recent analogous investigations on hydrated cordierite^13^ have revealed an order-disorder ferroelectric phase transition of the water molecules occurring below 3 K.

The fact that water dipoles may contribute to ferroelectric ordering has been known since the discovery of the first ferroelectric crystal Rochelle salt ($\hbox {NaKC}_4\hbox {H}_4\hbox {O}_6 \cdot 4\hbox {H}_2\hbox {O}$) a century ago^14^. Its ferroelectric phase (monoclinic space group $P2_1$) exists only between 255 K and 297 K, in contrast to all other ferroelectrics. Outside this temperature interval, Rochelle salt is paraelectric with orthorhombic symmetry (space group $P2_12_12$). Older neutron diffraction data^15^ showed that in the paraelectric phases, the whole tartrate and crystal water molecules were disordered with a small disordering amplitude. A newer reinvestigation in the paraelectric phase using synchrotron X-ray diffraction^16^ revealed a disorder only among K atoms and three O atoms of water, whereas the bonded H atoms of water are ordered. This shows that water molecules play a key role in the ferroelectricity of Rochelle salt. Despite these observations, the general understanding of the involvement of water molecules in water-ordering phenomena remains incomplete, still requiring further studies, both experimental and theoretical, of crystals containing isolated water molecules, such as lawsonite.

Because of the dipole moment of water molecules, and owing to their involvement in the collective polar crystal excitations, the relevant information on the internal crystal dynamics directly enters into the frequency-dependent complex dielectric permittivity $\hat{\varepsilon }(f)$ of these materials. Consequently, it appears useful to perform permittivity measurements by means of the methods of the dielectric spectroscopy. Similarly to the case of water molecules in the three main phases which show an electromagnetic response over a broad range of frequencies^17^, the interactions between the crystal water and the remaining crystal lattice can, in general, determine the dynamical crystal properties over many frequency decades. In order to obtain as complete information as possible about the lattice dynamics of lawsonite, including its ferroelectric properties, we undertook a study aiming at acquiring and analyzing its permittivity spectra over a broad range of frequencies by several experimental techniques.

## Properties of lawsonite

Lawsonite is a crystal with a very stable structure and a Mohs hardness^2^ of 7.5. From the crystallographic point of view, it can be viewed as a framework of corner- and edge-sharing $\hbox {SiO}_4$ tetrahedra and $\hbox {AlO}_6$ octahedra^18–21^. A small part of the oxygen atoms pertain to one polyhedron only, forming, together with the nearby hydrogen atoms, either hydroxyl groups, or water molecules. Thus, lawsonite contains 11.5 wt.% of water which is stoichiometrically distributed (one molecule per formula unit) across the lattice, and its presence is crucial for the crystal stability. Only upon heating above $\approx 850$ K, dehydration and breakdown of the structure occur^3^. Lawsonite crystals are mostly translucent and white^2^; a blue or pink coloring indicates substitution of Al atoms by Fe or Cr.

At room temperature, lawsonite is orthorhombic (space group *Cmcm*) with four formula units in the conventional (*c*-face centered) unit cell; the lattice parameters amount^19^ to $a=5.850\,\AA$, $b=8.790\,\AA$ and $c=13.122\,\AA$. (Note that we adhere to the standard convention $a<b<c$, which was, though, not followed by a few older publications.) The $\hbox {H}^{+}$ ions (protons) contained both in the water molecules and in the hydroxyl groups are interconnected by hydrogen bonds, forming quasi-one-dimensional chains parallel to the *c* axis^21^. The protons may diffuse along these chains^22^, especially above room temperature, and their configurations in the crystal lattice change at the two known low-temperature phase transitions. First, at $T_{\mathrm{c}1}=273$ K, due to a phonon instability at the Brillouin zone boundary, the crystal undergoes an antiferrodistortive structural phase transition to the *Pmcn* space group symmetry^20^. This loss of mirror symmetry is associated with rotations of both the hydroxyl groups and the water molecules within the *bc* plane^22^. Whereas the primitive cell volume is doubled, the resulting unit cell of the Bravais lattice coincides with the room-temperature conventional unit cell. Below $T_{\mathrm{c}1}$, the lattice parameter *a* shows an anomalous expansion upon cooling down to about 40 K^18,19^, which indicates a strong coupling between the order parameter and the strain tensor^23^. Another phase transition to a ferroelectric phase occurs at $T_{\mathrm{c}2}=124$ K, as the protons are displaced also along the *a* axis^22^. The orthorhombic symmetry persists down to the low-temperature ferroelectric phase which pertains to the $P2_1cn$ space group^24,25^. The spontaneous polarization vector was reported to point along the *a* axis, reaching up to $P_{\mathrm{s}} =0.3\,\upmu \mathrm{C}/\mathrm{cm}^2$ at 40 K^26^.

The phase transitions in lawsonite have attracted a considerable interest of numerous earlier works. The structures of the individual phases were studied experimentally using X-ray diffraction^18–20^ and neutron scattering^21^. At the phase transitions, other techniques have detected related anomalies, including those in specific heat^25,27^, thermal expansion^25^, resonant ultrasound measurements^28,29^, and in optical birefringence^23^. A number of studies concentrated on the ferroelectric phase transition; based on methods of dielectric spectroscopy, hysteresis loops of a polycrystalline sample^24^ and frequency-dependent complex permittivity $\varepsilon _a$ were measured, showing a peak near $T_{\mathrm{c}2}$^23,26^. Hayward et al.^27^ studied the effect of deuteration on the phase transitions. They found that replacing hydrogen by deuterium has no influence on the value of $T_{\mathrm{c}1}$, from which they concluded that the polyhedra distortion is the primary mechanism of the antiferrodistortive phase transition. In contrast, deuteration increases the value of $T_{\mathrm{c}2}$ by about 13 K, indicating that the proton ordering drives the ferroelectric phase transition.

In Ref.^23^, Sondergeld et al. interpreted the ferroelectric phase transition as a proper one. They presented their results as a confirmation of the order-disorder type of the phase transition, which had been suggested earlier by Libowitzky and Rossman based on mid-infrared (IR) spectroscopy^22^, sensitive to the O–H stretching modes. Despite that, there is still a controversy about the driving mechanisms and types of the two phase transitions, especially of the ferroelectric one. Whereas Salje et al. argued^24^, based on the fact that the polarization below $T_{\mathrm{c}2}$ is linearly temperature dependent, that this phase transition is an improper ferroelectric one, more recently, Pavlov and Romanov^30^ provided a phenomenological description of the anomalies in entropy and specific heat, supporting the proper-ferroelectric scenario. Meyer et al.^19^ studied the phase transitions, based on temperature-dependent IR powder absorption spectra and X-ray powder diffraction, from the standpoint of the Landau theory. They found indications that instead of the proton ordering, the driving mechanism could be some lattice distortion, presumably including a soft mode. Nevertheless, there has not been any report of a soft mode in lawsonite yet, and the complex nature of the two phase transitions, including the role of the water molecules, is still unresolved.

In the present work, we studied experimentally a single crystal of lawsonite by a set of methods of broadband dielectric spectroscopy. Polarized spectra of complex permittivity were measured in the frequency interval from 1 Hz to 20 THz, at temperatures ranging from 10 to 500 K. Several anomalies linked to the two phase transitions were observed. Surprisingly, we found that although the low-frequency dielectric anomaly at $T_{\mathrm{c}2}$ is caused by a critical slowing down of a dielectric relaxation, which is typical of order-disorder phase transitions, there is also an optical soft mode in the spectra polarized perpendicularly to the ferroelectric polarization.

## Methods

A colorless single crystal of lawsonite with dimensions of about $4\times 2\times 2\,\hbox {mm}^3$ was obtained by courtesy of Dr. Kozlova from Novosibirsk, Russia. The sample originating from Reed Ranch, Tiburon peninsula, California, had been provided to her by Prof. Armbruster, and it had been studied also in Ref.^31^. First, we verified the crystal orientation and purity by X-ray scattering; all reflections were identified in agreement with the structural data from Ref.^18^. Later, by subsequent polishing and cutting the crystal along the crystallographic axes, several plane-parallel slabs were obtained, allowing for measuring the three complex components of the dielectric permittivity $\hat{\varepsilon }_a$, $\hat{\varepsilon }_b$, $\hat{\varepsilon }_c$ by various techniques. The application of the spectroscopic techniques was partly limited by the relatively small size of the original crystal.

Low-frequency (1 Hz–1 MHz) dielectric measurements at temperatures between 10 and 300 K were performed using a Novocontrol Alpha-A impedance analyzer with the samples mounted in a Janis He-flow cryostat. For these measurements, opposite surfaces of the samples were covered with electrically conducting silver paste serving as electrodes. THz time-domain spectroscopy was performed using a custom-made setup in the transmission geometry, within the frequency interval of about 0.25–2 THz; the upper limit was influenced by the absorption in the crystal. For temperature stabilization within the range of $T=20$–500 K, we used a He-flow optical cryostat (Optistat, Oxford Instruments) and a high-temperature cell (Specac). Further details about the THz spectroscopy technique and data treatment are described in Ref.^32^. Polarized IR reflectivity spectra were measured using the Fourier-transform spectrometer Bruker IFS113v in the frequency interval of about 1.5–20 THz (50–660 $\hbox {cm}^{-1}$), at temperatures from 10 K to 500 K.

## Results and discussion

**Figure 1:**
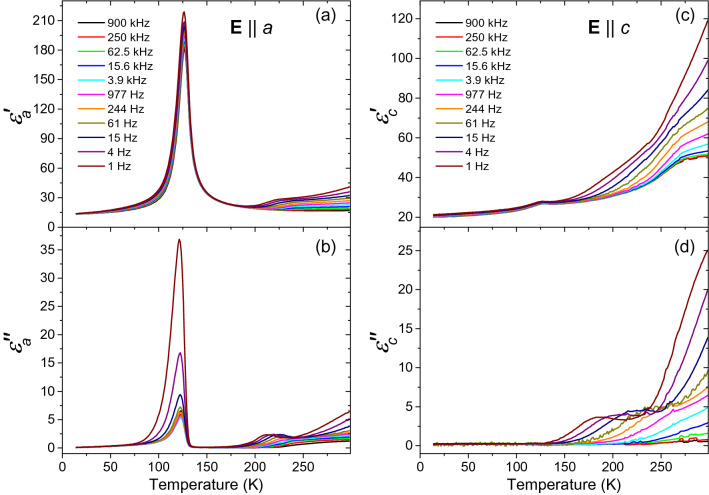
Temperature dependences of the complex permittivity $\hat{\varepsilon }_a$, $\hat{\varepsilon }_c$ at selected frequencies. Panels (**a**,**c**): real parts, panels (**b**,**d**): imaginary parts.

Figure 1 shows temperature dependences of the complex permittivity at selected frequencies within the range of 1 Hz–0.9 MHz, using electric field orientations $\mathbf {E}\parallel a$, $\mathbf {E}\parallel c$. The orientation $\mathbf {E}\parallel b$ is missing, because the size and shape of the available sample did not allow for reliably measuring in this geometry. In the $\hat{\varepsilon }_a$ component, we detected the anomaly at $T_{\mathrm{c}2}=124\,K$ reported by Sondergeld et al.^23,26^. We note that the absolute values of this permittivity component are very different, though. Whereas Sondergeld et al. reported a peak value of $\hat{\varepsilon }_a\approx 800+300\,\mathrm{i}$ at $f=10$ kHz^23,26^, our measurements yielded $\hat{\varepsilon }_a\approx 190+5.3\,\mathrm{i}$ at this frequency. The reason of this discrepancy is unclear; obviously, different samples were used in the two measurements. It is evident that the mismatch cannot be accounted for by a simple scaling factor, as the loss tangent (i.e., the $\varepsilon _a^{\prime \prime }/\varepsilon _a^{\prime }$ ratio) values obtained are also very different—we obtained $\tan \delta \approx 0.03$, more than an order of magnitude lower than reported by Sondergeld et al.. In contrast, the $\hat{\varepsilon }_c$ component [Fig. 1c,d] shows only a very weak anomaly at $T_{\mathrm{c}2}$, which we will not discuss here because it might be merely due to a leakage from the *a* direction. A slightly stronger broad anomaly for $\mathbf {E}\parallel c$ was found near $T_{\mathrm{c}1}$, however, it is accompanied by a continuous increase occurring in both real $\varepsilon _c^{\prime }(T)$ and imaginary $\varepsilon _c^{\prime \prime }(T)$ parts. This increase sets on near $T=150$ K, and it further enhances with temperature; it can be most probably attributed to an onset of proton hopping along the paths connecting the crystallographic sites of hydrogen atoms (see also Fig. 12 in Ref.^21^), in conjunction with a crystal inhomogeneity on the microscopic scale in this direction, preventing dc conduction. This manifests itself by a Maxwell-Wagner dielectric relaxation in the impedance spectra.

**Figure 2:**
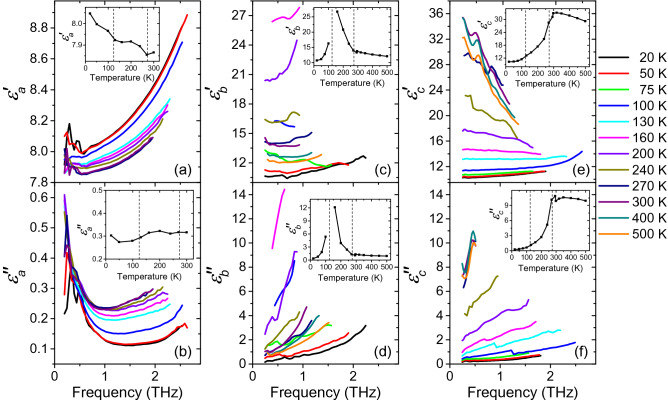
Polarized spectra of complex permittivity of lawsonite in the THz range as a function of temperature. Panels (**a**,**c**,**e**): real parts, panels (**b**,**d**,**f**): imaginary parts. The insets show temperature dependences of the appropriate values at the frequency of $f=0.5$ THz; points: measured values, lines: guides for the eye.

Spectra of complex permittivity obtained from the THz measurements are drawn in Fig. 2. Their shapes markedly depend on both temperature and polarization; the spectra are consistent with those of IR reflectivity measured in the adjacent frequency interval. As discussed below, our fits revealed that the increases in $\varepsilon _a^{\prime \prime }(f)$ and $\varepsilon _c^{\prime \prime }(f)$ toward the low-frequency limits of the THz spectra can be explained by the presence of relaxation terms whose relaxation frequencies are located in the GHz range. The increases in all the three components of $\varepsilon ^{\prime \prime }(f)$ toward the high frequencies are due to phonons with frequencies beyond 2 THz. Interestingly, the spectra follow quite an unexpected temperature evolution, which can be seen in the insets of Fig. 2, displaying the values of permittivity at 0.5 THz. In fact, the $\varepsilon _a^{\prime }$ component shows a monotonic decrease, without any anomalies around the phase transitions temperatures. This testifies an absence of a soft polar phonon in the $\mathbf {E}\parallel a$ polarized spectra. In contrast, a pronounced increase in the $\hat{\varepsilon }_b$ component corresponding to a maximum at $T_{\mathrm{c}2}$ was observed, due to which the sample became opaque near the ferroelectric phase transition. This indicates a polar phonon exhibiting softening near $T_{\mathrm{c}2}$. Finally, both the real and imaginary parts of the $\hat{\varepsilon }_c$ component of the permittivity increased with temperature up to $T_{\mathrm{c}1}$ where a gradual change in their slopes was detected. Again, this indicates a phonon anomaly in the $\mathbf {E}\parallel c$ spectra, occurring near the antiferrodistortive phase transition temperature $T_{\mathrm{c}1}$. These observations are discussed in more detail below. Let us also note that the values of $\varepsilon _a^{\prime }$ in the THz range [shown in Fig. 2a] are systematically markedly lower than those provided near 1 MHz [see Fig. 1a]. These temperature-dependent offsets (amounting to 7 at 20 K) can be explained by the presence of a soft dielectric relaxation in the high-frequency (MHz to GHz) range; this relaxation appears to play a key role in the ferroelectric phase transition. Similar offsets (amounting to 10 at 20 K) between the THz and MHz-range values were observed also in the $\varepsilon _c^{\prime }$ component [see Figs. 1c, 2e].

**Figure 3:**
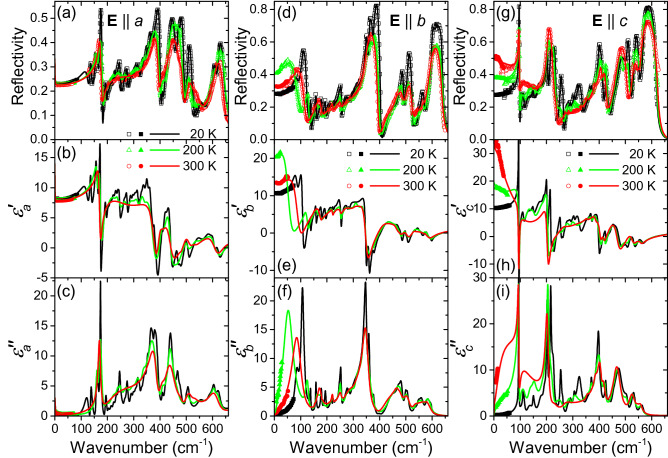
Comparison of polarized reflectance data and complex permittivity spectra of lawsonite in the IR range at 20, 200 and 300 K. Symbols below and above $100\,\hbox {cm}^{-1}$: experimental data from THz time-domain transmittance [calculated from the complex permittivity using Eq. (2)] and IR reflectivity measurements, respectively. Lines: spectra fits.

Polarized IR reflectivity spectra measured as a function of temperature are shown in Fig. 3. Depending on the spectra polarization and crystal symmetries, we observed different numbers of vibrational modes which are assigned to transverse polar phonons. The IR spectra were fit together with the THz spectra using the factorized model of permittivity, expressed by the formula^33^:

1 $$\begin{aligned} \hat{\varepsilon }(f) \equiv \varepsilon ^{\prime }(f) + \text{ i } \varepsilon ^{\prime \prime }(f) = \varepsilon _\infty \prod _{j=1}^N \frac{f_{\mathrm{LO}j}^2-f^2+\mathrm{i}f\gamma _{\mathrm{LO}j}}{f_{\mathrm{TO}j}^2-f^2+\mathrm{i}f\gamma _{\mathrm{TO}j}} \end{aligned}$$

where $\varepsilon _\infty$ is the high-frequency (optical or near-IR) background value of permittivity, $f_{\mathrm{LO}j}$ and $f_{\mathrm{TO}j}$ denote the optical phonon frequencies, $\gamma _{\mathrm{LO}j}$ and $\gamma _{\mathrm{TO}j}$ are the damping constants of the *j*-th longitudinal and transverse phonon modes, respectively. From the dielectric function, the reflectance was calculated using the Fresnel formula for normal incidence:

2 $$\begin{aligned} R(f)= \left| \frac{\sqrt{\hat{\varepsilon }(f)}-1}{\sqrt{\hat{\varepsilon }(f)+1}}\right| ^2\,. \end{aligned}$$

**Figure 4:**
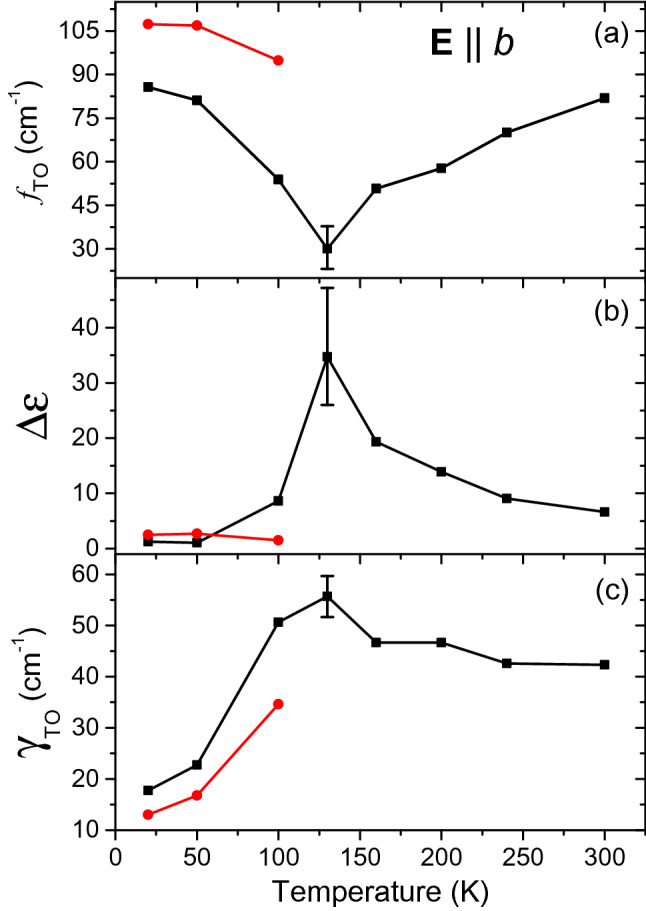
Temperature dependence of oscillator parameters characterizing the soft phonon in the $\mathbf {E}\parallel b$ polarization, obtained by fitting the IR reflectivity spectra using Eqs. (1), (2). (**a**) oscillator frequency, (**b**) oscillator strength $\Delta \varepsilon$, (**c**) oscillator damping $\gamma _{\mathrm{TO}}$. Black symbols: dominant phonon, red: weaker mode arising below $T_{\mathrm{c}2}$ due to symmetry lowering. The lines are guides for the eye only. Errors in determining the parameters are approximately equal to the symbol size, except at $T=130$ K where they are shown by the error bars; note that no THz data was available for that temperature.

For all observed phonons, this model provided a good agreement with the experimental data. It is worth noting that a minor part of the phonon frequencies display an anomalous behavior, as they weakly decrease with temperature. This is presumably linked to the anomalous small increase in the lattice parameter *a* upon cooling. In the $\mathbf {E}\parallel b$ geometry, the lowest-frequency phonon exhibits an anomalous temperature behavior [see Fig. 3d–f]. The mode parameters obtained from the fits as a function of temperature are shown in Fig. 4. Here the phonon dielectric strength was evaluated using the formula^33^

3 $$\begin{aligned} \Delta \varepsilon _{bj}(f)= \varepsilon _\infty f^{-2}_{\mathrm{TO}j} \frac{\prod _k f^2_{\mathrm{LO}k} -f^2_{\mathrm{TO}j}}{\prod _{k\ne j}f^2_{\mathrm{TO}k} -f^2_{\mathrm{TO}j}}\,. \end{aligned}$$

The phonon frequency displays a clear minimum of about $30\,\hbox {cm}^{-1}$ at $T=130\,\mathrm{K}$, where a peak value of $\Delta \varepsilon _b \approx 35$ is observed. An additional phonon near $100\,\hbox {cm}^{-1}$ is activated in the ferroelectric phase, and it displays also a weak frequency rise on cooling; its parameters are shown by the red symbols in Fig. 4. Nevertheless, the dielectric strength of this additional mode is substantially lower. Consequently, the above-described behavior is typical of a soft phonon associated with the ferroelectric phase transition, despite the fact that its dynamic polarization ($\mathbf {E}\parallel b$) is perpendicular to the ferroelectric axis *a*. At the same time, we note the relatively high value of its minimum frequency, about $30\,\hbox {cm}^{-1}$ near $T_{\mathrm{c}2}$, which is in agreement with the fact that the soft phonon does not cause lowering of the crystal symmetry in the ferroelectric phase. To our knowledge, this is the first known material where an order-disorder phase transition (which is discussed below) is associated with a soft polar phonon whose polarization is perpendicular to the ferroelectric axis.

Further, in the $\mathbf {E} \parallel c$ geometry, a special shape of the IR spectra was observed around $100\,\hbox {cm}^{-1}$ at all temperatures; this feature consists of a sharp peak and a broad reflectivity background, spanning over more than $100\,\hbox {cm}^{-1}$. This shape of the IR spectra can be well described by the model given by Eq. (3) which is inherently suitable for describing the dynamical interaction between these two vibrational modes. Complex IR permittivity spectra calculated from the fits at various temperatures, together with a table of phonon parameters at 20 and 300 K, can be found in the Supplementary material online (Fig. S3 and Tab. S3). In the low-frequency part of the IR spectra, namely below $\approx 80\,\hbox {cm}^{-1}$, a broad mode is present, matching well the shape of the THz spectra of $\hat{\varepsilon }_c$. Similarly to these, the low-frequency reflectivity along the *c* axis attains its minimum value of about 0.30 near $T=20$ K (see Fig. 3g). Upon heating, it rises monotonically, attaining a flat maximum of about 0.5 at $T=270$–300 K, and it decreases again on heating above room temperature [see also Supplementary Fig. S1(c)]. This temperature dependence, obvious also from the THz permittivity (see insets of Fig. 2e,f), is due to an overdamped excitation whose dielectric strength decreases on cooling, until it vanishes below $T_{\mathrm{c}2}$. This provides an evidence that this excitation does not correspond to a phonon. Instead, it is most probably due to the hopping of protons at higher temperatures which gradually disappears on cooling (see also Supplementary Fig. S6 online).

The numbers of modes permitted in the polarized IR and Raman spectra of the individual phases of lawsonite were calculated using the factor-group analysis, whose results are summarized in Table 1. The numbers of modes contained in our spectra fits are not in contradiction with the factor-group analysis. Up to 37, 33 and 29 phonons were identified in the lowest-temperature (20 K) far-IR spectra in the $\mathbf {E} \parallel a$, $\mathbf {E} \parallel b$, and $\mathbf {E} \parallel c$ polarizations, respectively. All values of phonon parameters found by fitting in the three polarizations and phases can be found in Supplementary Tabs. S1–S3 online.

**Table 1:**
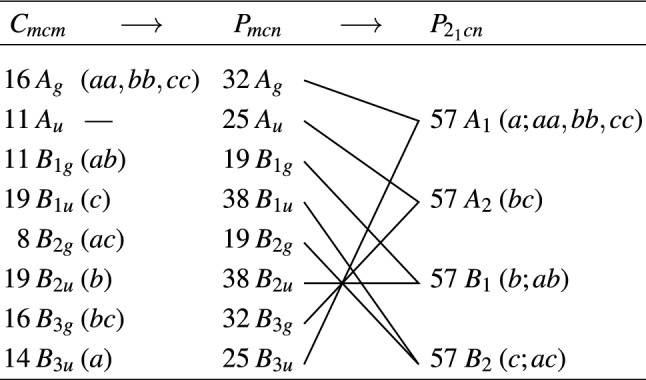
Factor group analysis summarizing activities of phonon modes in the three phases of lawsonite (including acoustic modes).

**Figure 5:**
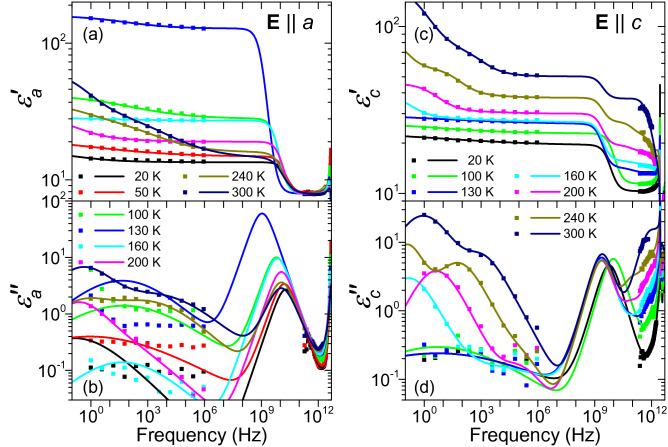
Broadband fits of complex permittivity $\hat{\varepsilon }_a$ (**a**,**b**) and $\hat{\varepsilon }_c$ (**c**,**d**) of lawsonite as a function of temperature. Symbols: experimental values obtained by impedance (selected frequencies) and THz spectroscopies; lines: fits using Eq. (4). The estimated precision of the complex permittivity values is $\pm 3\%$.

**Figure 6:**
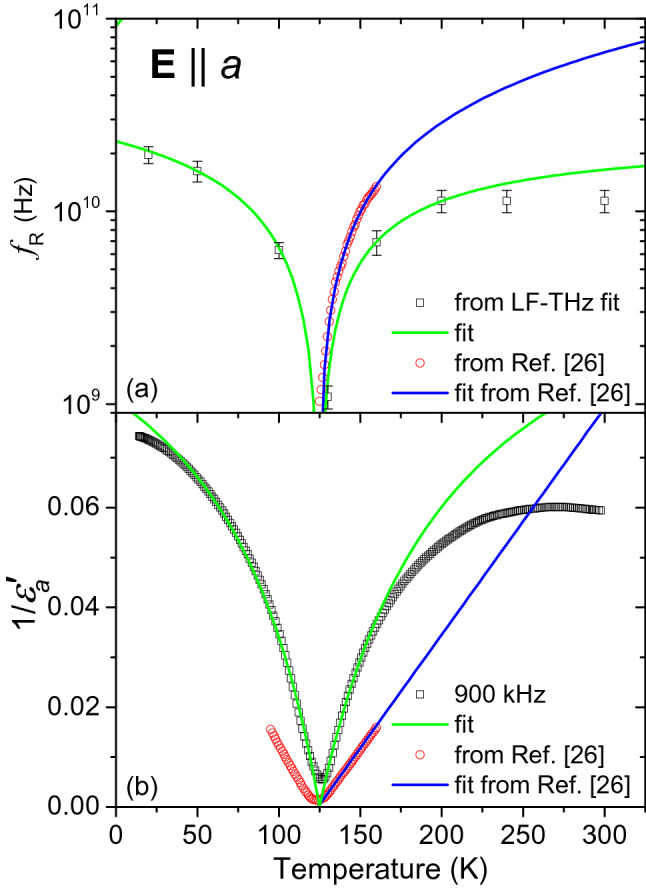
Anomaly in the $\varepsilon _a$ permittivity component at $T_{\mathrm{c}2}$ due to the soft Debye relaxation behavior. Panel (**a**): Temperature dependence of the relaxation frequency $f_{\mathrm{r}}$ (symbols) and its fits using Eq. (5) (green lines). For sake of comparison, values from Ref.^26^ are shown. Panel (**b**): reciprocal real part of permittivity $1/\varepsilon _a^{\prime }$—experimental values (symbols) and fits using the Curie-Weiss law (lines). The estimated precision of our reciprocal permittivity values is $\pm 3\%$.

In order to obtain an overall picture of the dynamical processes in lawsonite related to the phase transitions, we performed broadband fitting of its dielectric response within the interval from 1 Hz to 3 THz, covering the measured impedance and THz ranges. To this aim, we used the Cole-Cole formula:

4 $$\begin{aligned} \hat{\varepsilon }(f)=\sum _j\frac{\Delta \varepsilon _j}{1+ (\mathrm{i} f/f_{\mathrm{r}j})^{1-\alpha _j}}+\hat{\varepsilon }^{\mathrm{IR}}(f) \end{aligned}$$

where $\Delta \varepsilon _j$ denotes the dielectric strength of the *j*-th term, $f_{\mathrm{r}j}$ its relaxation frequency, and $0\le \alpha _j\le 1$ is the Cole-Cole parameter. Depending on temperature, up to three and four relaxations were used in the $\mathbf {E}\parallel a$ and $\mathbf {E}\parallel c$ polarizations, respectively. For the special case of $\alpha _j=0$, the Cole-Cole formula is identical with that describing the Debye relaxation. The function $\hat{\varepsilon }^{\mathrm{IR}}(f)$ is given by Eq. (1)—it consists of contributions of all IR phonons with the parameters and numbers of modes provided by the fits of IR spectra. The resulting complex permittivity spectra $\hat{\varepsilon }_a(f)$, $\hat{\varepsilon }_c(f)$ for selected temperatures are shown by lines in Fig. 5. In both these permittivity components, there are mostly marked offsets between the values of the real parts in the THz range and those just below 1 MHz, see Fig. 5a,c. Consequently, it was necessary to include the Cole-Cole formula into the model, in order to correctly describe the spectra, although our experimental techniques did not completely cover the appropriate spectral range. In fact, the limited volume of the sample available was insufficient for performing measurements by microwave resonance techniques. In the spectra of permittivity $\hat{\varepsilon }_a(f)$, corresponding to the direction of ferroelectric polarization, fitting provided a Debye term with a room-temperature relaxation frequency of about 10 GHz, see Fig. 5a,b. As the temperature was decreased toward $T_{\mathrm{c}2}$, a drop in $f_{\mathrm{r}}$ was observed along with the increase in $\varepsilon _a^{\prime }$. We found that the temperature dependence of this Debye term can be described well by the critical slowing-down formula^34^:

5 $$\begin{aligned} f_{\mathrm{r}}^{-1}(T)= \left[ A(T-T_{\mathrm{c}})\right] ^{-1} +f_{\mathrm{s}}^{-1} \end{aligned}$$

where $f_{\mathrm{r}}$ and $f_{\mathrm{s}}$ denote the soft relaxation frequency and its asymptotic high-temperature value, respectively, and *A* is a free fitting parameter. The temperature dependence of the relaxation frequency $f_{\mathrm{r}}(T)$ is shown in Fig. 6a. The fits of our data yielded these parameter values: $A=(2.8\pm 0.3)\times 10^8\,(\mathrm{s}\,\mathrm{K})^{-1}$ in both phases, $f_{\mathrm{s}}=(2.5 \pm 0.5)\times 10^{10}\,\mathrm{s}^{-1}$ and $f_{\mathrm{s}}=(6.4 \pm 0.5)\times 10^{10}\,\mathrm{s}^{-1}$ in the paraelectric and ferroelectric phases, respectively. The ferroelectric phase transition near $T_{\mathrm{c}2}$ is thus of an order-disorder type.

In Fig. 6b we plot the temperature dependence of the reciprocal value of the permittivity $1/\varepsilon _a^{\prime }(T)$ at $f\approx 900$ kHz, i.e., below the critical relaxation frequency. This data is compared with that by Sondergeld et al.^26^. In the temperature interval spanning over several tens of kelvins from $T_{\mathrm{c}2}$, both these datasets follow the Curie-Weiss law, $1/\varepsilon _a^{\prime }(T)= \left[ C/(T-T_{\mathrm{c}2}) +\varepsilon _a^{\prime \mathrm{THz}} \right] ^{-1}$, with Curie constant values of $C = 650\pm 50$ K (our data) and $C \approx 2200$ K (Ref.^26^), respectively. Note that our fit is consistent with the high-frequency (THz-range) permittivity value of $\varepsilon _a^{\prime \mathrm{THz}}\approx 8$ (cf. Fig. 2a). In contrast, Sondergeld et al. performed the fit in a narrower temperature range (within $\pm 30$ K from $T_{\mathrm{c}2}$); also, their data were obtained in a more restricted frequency range compared to our measurements. The relatively low value of *C* and a rather narrow temperature range of the dielectric anomaly are consistent with the ferroelectric transition of the pseudoproper type^35^ where the polarization is not the order parameter; however, the order parameter and the polarization have the same symmetry and are bilinearly coupled. In pseudoproper ferroelectrics, the predominant atomic displacements in the order parameter do not contribute to the polarization. This kind of phase transitions were also later independently named weak ferroelectric transitions^36^. Let us stress that they differ from the improper ferroelectric ones where the order-parameter symmetry also differs from that of the polarization. Unlike the data published in Ref.^24^, our pyrocurrent measurements revealed an increase below $T_{\mathrm{c}2}$ which is linear only in a narrow temperature interval, within about 110–120 K (see Supplementary Fig. S4 online). Nevertheless, the temperature dependence of the spontaneous polarization in lawsonite below the ferroelectric phase transition can still resemble that of improper ferroelectrics, as revealed in Ref.^25^. Moreover, the unusual spectral feature linked to this phase transition is the presence of a soft mode with an eigenmode orthogonal to the spontaneous polarization, in the polarization $\mathbf {E}\parallel b$.

The most pronounced spectral feature accompanying the antiferrodistortive phase transition near $T_{\mathrm{c}1}$ is the flat maximum in permittivity $\hat{\varepsilon }_c$ in the THz range, see Fig. 2e,f, which extends also to the lowest-frequency part of the IR spectra (see Fig. 3g–i). The broadband fits (see Fig. 5c,d) revealed an overdamped oscillator in the far-IR range. Its frequency is almost temperature-independent, but its damping strongly increases on cooling below $T_{\mathrm{c}1}$. For that reason, the frequency of its peak in dielectric loss—located at $f_{\mathrm{TO}}^2/\gamma _{\mathrm{TO}}(T)$—decreases, and also $\Delta \varepsilon (T)$ of this excitation drops on cooling, see Supplementary Fig. S6 online. We note that this critical oscillation has characteristic frequencies about three orders of magnitude higher than the one inducing the ferroelectric phase transition [see Fig. 6]. This appears to be in line with the earlier hypothesis by Hayward et al.^27^ claiming that the antiferrodistortive phase transition is driven by motions of some structure polyhedra, whereas the ferroelectric one occurs due to proton ordering. The characteristic frequency of proton relaxation ($\approx 1$ GHz) in the $\hat{\varepsilon }_a$ component, pertaining to the ferroelectric phase transition, would then supposedly correspond to proton hopping between adjacent positions. In contrast, the THz-range frequency of the critical oscillation in the $\hat{\varepsilon }_c$ component, linked to the antiferrodistortive phase transition, might be linked to small proton displacements within their equilibrium potential minima.

## Conclusions and outlook

In summary, we performed a comprehensive study of lattice dynamics in a lawsonite single crystal by measuring its complex permittivity spectra over a broad range of temperatures and frequencies. In the temperature interval delimited by the two structural phase transitions, we observed a gradual decrease in the dielectric strength of the lowest-lying overdamped mode in the $\mathbf {E}\parallel c$ polarization upon cooling, accompanied by an increase in its damping. These changes were observed in the THz and far-IR spectra in the frequency interval from 0.25 THz to about 3 THz via a broad absorption which ultimately disappears below the ferroelectric phase transition at $T_{\mathrm{c}2}$; therefore we conjecture that this broad absorption is linked to the motions of disordered protons. The ferroelectric phase transition is further connected with two other anomalies. In the polarization of electric field along the ferroelectric axis *a*, we observed a critical slowing down of the relaxation frequency, typical of ferroelectric phase transitions of the order-disorder type. This slowing down produces a peak in the Hz-to-MHz permittivity $\varepsilon _a^{\prime }$ at $T_{\mathrm{c}2}$, qualitatively similar to the anomaly observed earlier^23,26^. In the orthogonal permittivity component $\hat{\varepsilon }_b$, a soft polar phonon, typical of displacive phase transitions, with a minimum frequency attained at $T_{\mathrm{c}2}$ leads to a small increase in both real and imaginary parts. Since the ferroelectric polarization is orthogonal to the *b* direction, this phonon anomaly appears to be limited to the THz and far-IR regions, without substantially influencing the lower-frequency dynamics.

The unexpected behavior consists in the fact that the dynamics seem to be coupled among the orthogonal directions in the crystal. We tentatively attribute this to the rotations of water molecules and perhaps also of the hydroxyl groups. Between the two phase transitions, the freezing of the motions of the protons along the *c* axis might influence the rotations of the hydroxyl groups or of the molecules of water, as they get ordered upon cooling toward the minimum of potential energy. This would qualitatively explain the soft mode observed along the *b* axis in the THz range. Finally, such rotational coupling could also result in the much slower lattice relaxation along the *a* axis at GHz frequencies, which we interpret as a characteristic of the order-disorder phase transition. All these hypotheses are only speculative, and they would need to be confirmed quantitatively by modeling. Nevertheless, the complexity of the crystal unit cell and the need for a detailed knowledge of the potential governing the proton motions make the modeling difficult to realize. Consequently, such a task seems greatly challenging for theoreticians. Alternatively, additional information about the proton dynamics might be also obtained experimentally by means of a temperature-dependent nuclear magnetic resonance study.

To the best of our knowledge, lawsonite is the only example of a crystal exhibiting a ferroelectric phase transition where two anomalies in two perpendicular directions coexist, even if only one of them is driving the symmetry lowering of the crystal structure. In fact, all other displacive ferroelectrics exhibit a soft phonon anomaly only in the THz spectra polarized along the ferroelectric axis. This is natural, because the transient dipole moment associated with the phonon is then parallel to the spontaneous polarization. In view of our observations in lawsonite, it will be interesting in future to search for other ferroelectrics exhibiting phonon and dielectric relaxation anomalies in two different crystallographic directions. Up to now, the dynamics of ferroelectrics phase transitions have been researched in much detail^34,37,38^ in numerous inorganic compounds (e.g., materials with the perovskite, Aurivillius or Ruddlesden-Popper structures). In contrast, the lattice dynamics of complex organic^39,40^ or organometallic^41,42^ ferroelectrics are rather poorly known, which makes them attractive for future studies of this kind, possibly providing an explanation of the unusual observed behavior.

## Supplementary information

Supplementary Information.

## Data Availability

The datasets used and/or analyzed during the current study are available from the corresponding author on reasonable request.
