# Supplementary material for: Unusual features of lattice dynamics in lawsonite near its phase transitions
Source: Sci Rep. 2022 Apr 13;12:6157. doi: 10.1038/s41598-022-09890-7 (PMC9008026; doi:10.1038/s41598-022-09890-7)
Supplement: Supplementary file 1 — Supplementary Information. [file 41598_2022_9890_MOESM1_ESM.pdf]

Supplementary material—Unusual features of lattice dynamics in lawsonite near its phase transitions

F. Kadlec[1],* D. Nuzhnyy[1], C. Kadlec[1], J. Petzelt[1], M. Savinov[1], and S. Kamba[1]
[1]*Institute of Physics, Czech Academy of Sciences, Na Slovance 2, 182 21 Prague 8, Czech Republic*

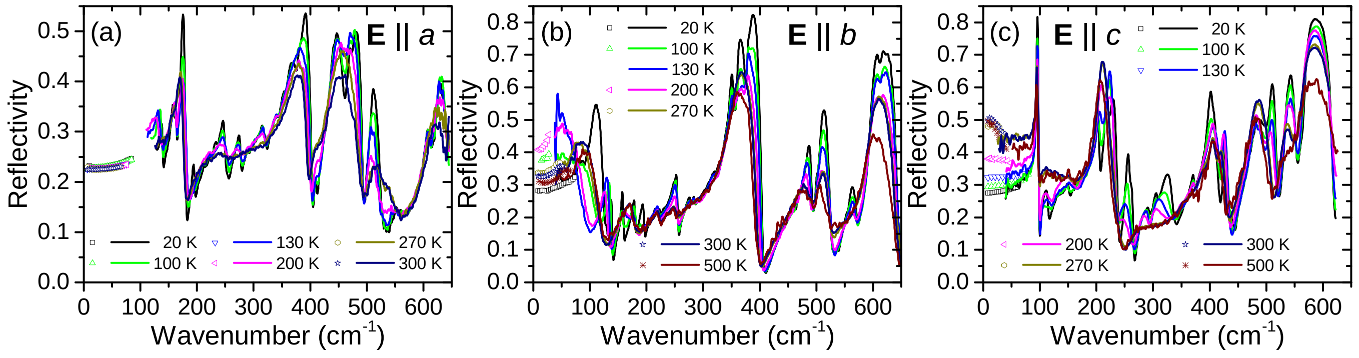

FIG. S1. Polarized IR reflectivity spectra (lines) measured at orientations of the electric field vector (a) $\mathbf{E} \parallel a$, (b) $\mathbf{E} \parallel b$, (c) $\mathbf{E} \parallel c$. Spectra in the panel (a) display an increased level of noise at low frequencies because of limited sample size available for this experimental geometry. Also, in this case, the sample had a lower thickness, so it was partly transparent at frequencies below $\approx 120\,\mathrm{cm}^{-1}$. Therefore, owing to an artificial increase in the measured signal, this data is not presented.

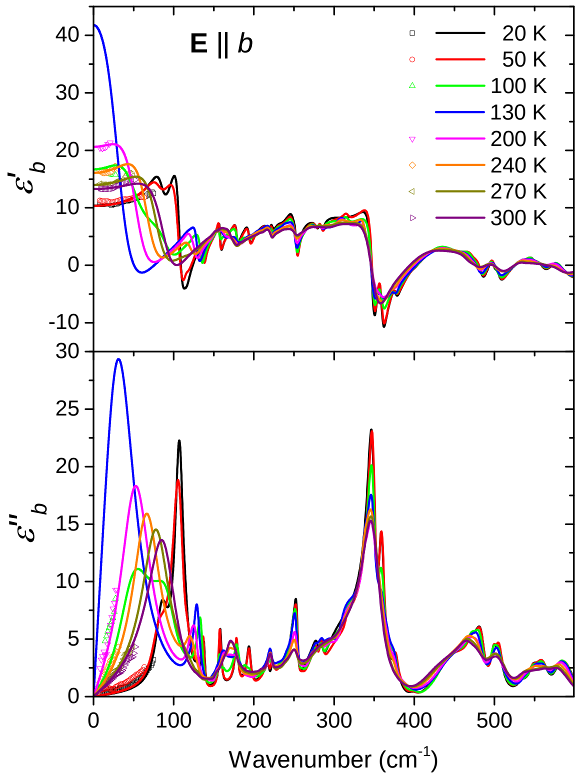

FIG. S2. Complex permittivity spectra $\varepsilon_b(f)$ of lawsonite in the infrared range at low temperatures obtained by fitting reflectivity spectra. Symbols show data obtained by THz spectroscopy.

* kadlecf@fzu.cz

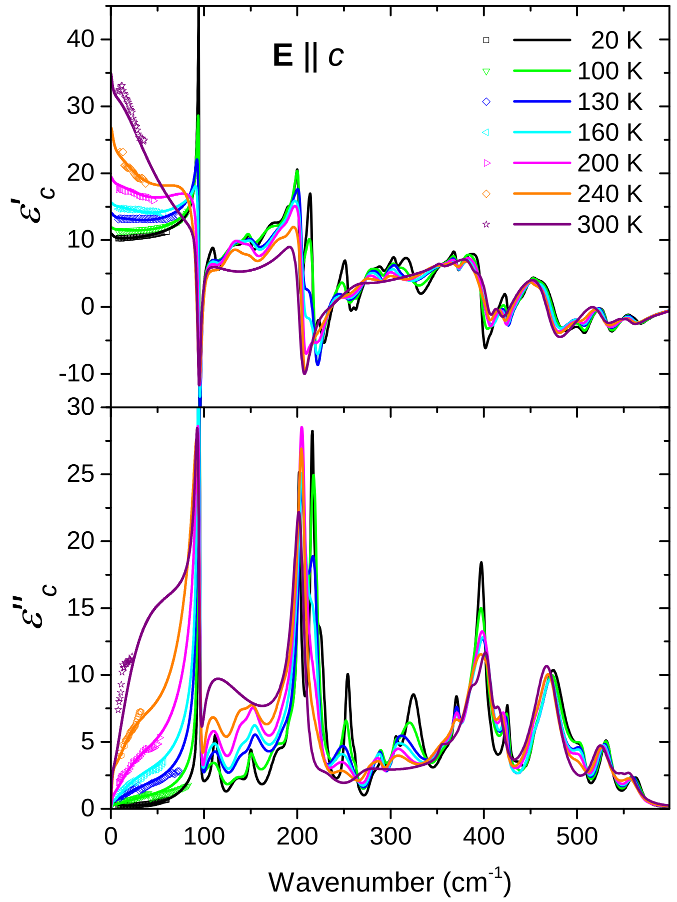

FIG. S3. Complex permittivity spectra $\varepsilon_c(f)$ of lawsonite in the infrared range at low temperatures obtained by fitting reflectivity spectra. Symbols show data obtained by THz spectroscopy.

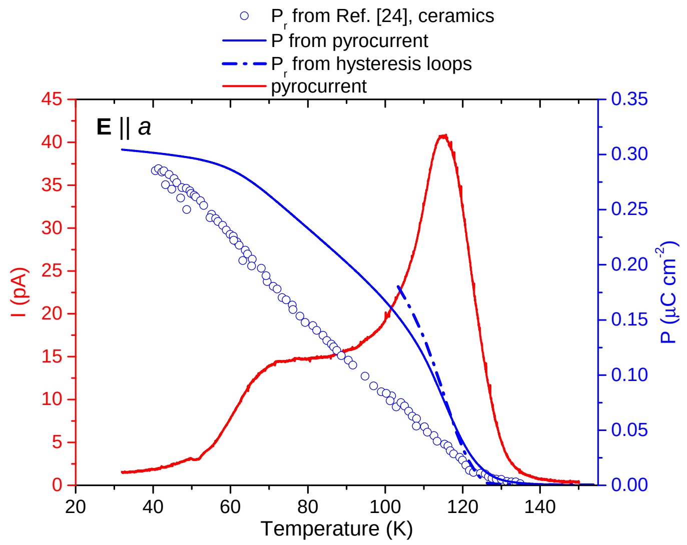

FIG. S4. Temperature dependence of the pyrocurrent $I(T)$ and remanent polarization $P_\mathrm{r}(T)$ measured along the ferroelectric a axis of lawsonite. Solid lines: Our results for pyrocurrent (red) and remanent polarization (blue) measured upon heating after cooling down in an electric field of $E = 2\,\mathrm{kV/cm}$. Dot-dashed blue line: $P_\mathrm{r}(T)$ calculated from hysteresis loops measured at peak electric field of $E = 5\,\mathrm{kV/cm}$. For comparison, $P_\mathrm{r}(T)$ data measured on ceramics by Salje *et al.* are shown by symbols (Ref. [24] from the main text).

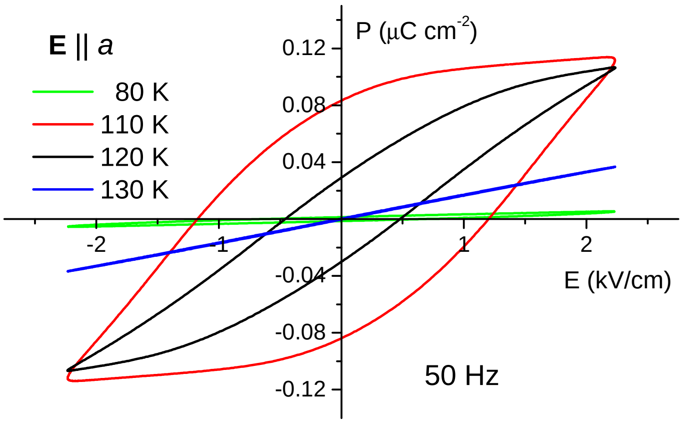

FIG. S5. Hysteresis loops measured using a peak electric field of $E = 2\,\mathrm{kV/cm}$ along the ferroelectric a axis.

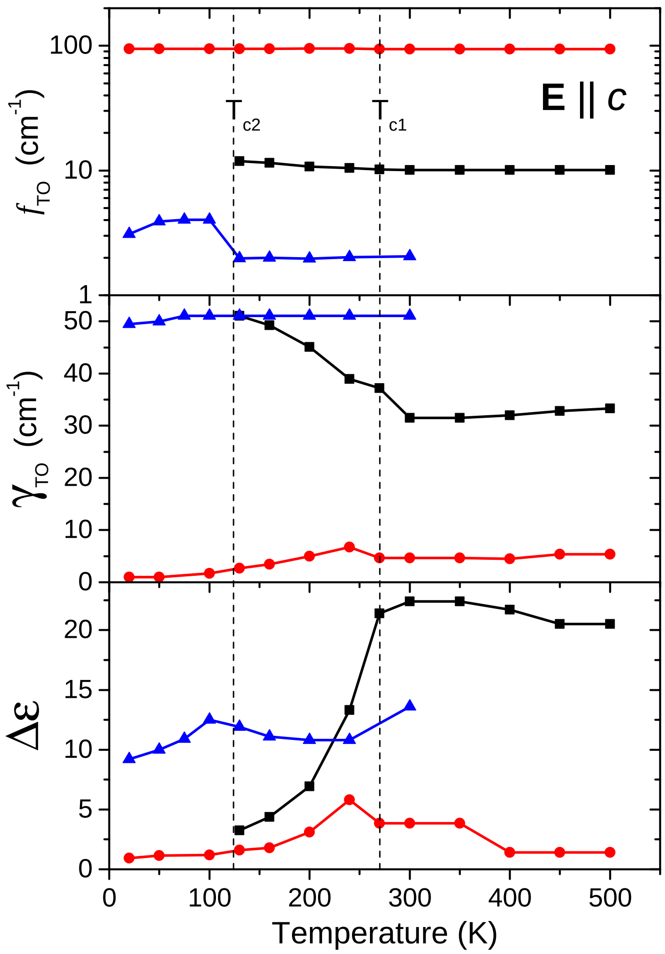

FIG. S6. Symbols: fit parameters describing the three lowest-frequency modes in the $\mathbf{E} \parallel c$ polarization, including the overdamped oscillator (black symbols) which is at the origin of the observed flat maximum in permittivity near T_{c1}. Lines are guides for the eye. The resulting spectra are shown in Fig. S3.

Table S1. Frequencies (f_{TOj}, f_{LOj}) and dampings (γ_{TOj}, γ_{LOj}) obtained from fitting polarized reflectivity spectra (**E**||**a**) in THz-IR range using Eqs. (1) and (2) with ε_∞=2.3.

Temperature	Number	f_{TOj} (cm^{-1})	γ_{TOj} (cm^{-1})	f_{LOj} (cm^{-1})	γ_{LOj} (cm^{-1})
**20 K**	1	3.8	27.3	5.1	28.4
	2	125.4	24.2	129.1	14.9
	3	136.7	7.0	139.6	11.1
	4	158.6	5.4	159.8	3.9
	5	161.6	4.7	163.7	7.6
	6	173.5	4.4	181.1	4.3
	7	193.8	7.5	196.9	7.1
	8	201.5	9.5	203.0	14.0
	9	212.3	12.5	214.4	16.4
	10	222.0	10.5	223.0	14.3
	11	232.0	11.8	233.0	17.9
	12	240.0	6.1	240.0	6.9
	13	246.2	7.0	248.4	10.2
	14	263.8	8.4	263.9	9.7
	15	275.0	7.2	277.0	7.8
	16	289.2	11.3	291.1	13.6
	17	296.9	8.7	297.0	10.5
	18	306.8	11.9	307.8	12.9
	19	315.8	8.6	317.9	7.3
	20	319.4	5.9	319.9	9.3
	21	327.9	8.0	328.0	9.2
	22	339.0	14.7	341.5	17.8
	23	351.6	10.5	352.8	13.6
	24	364.8	15.2	369.9	21.4
	25	372.0	6.8	372.3	7.5
	26	378.9	6.8	379.0	8.0
	27	388.0	19.4	402.8	7.9
	28	408.7	6.5	410.4	8.0
	29	421.2	12.3	423.0	14.7
	30	439.3	20.1	462.1	20.9
	31	467.4	9.6	471.0	14.3
	32	477.0	16.7	492.7	11.6
	33	500.0	7.2	501.0	8.0
	34	506.1	12.6	524.4	22.4
	35	550.6	37.1	565.1	48.7
	36	586.7	23.1	589.1	29.2
	37	606.1	14.3	610.8	15.5
	38	623.0	20.9	643.3	30.6

**200 K**	1	3.8	27.3	5.1	28.4
	2	161.3	7.1	162.3	5.9
	3	173.7	13.1	182.4	8.5
	4	193.4	18.4	195.5	20.6
	5	221.0	44.2	231.5	51.9
	6	239.4	12.4	241.0	13.3
	7	245.8	10.7	246.1	14.3
	8	268.9	21.6	271.5	25.3
	9	293.0	29.3	295.0	33.2
	10	316.0	16.5	317.6	16.8
	11	336.7	17.5	338.0	19.2
	12	375.0	48.7	400.0	12.0
	13	406.5	17.8	415.2	25.6
	14	436.4	28.5	463.0	23.8
	15	464.0	21.5	491.5	20.1
	16	501.2	20.2	527.4	29.3
	17	534.7	29.3	537.0	77.3
	18	608.0	10.5	609.0	10.0
	19	618.0	40.7	649.3	30.6
**300 K**	1	3.8	27.3	5.1	27.9
	2	170.5	11.7	177.5	9.5
	3	238.2	171.4	278.9	236.7
	4	385.9	39.2	393.9	12.9
	5	402.4	32.9	412.6	33.1
	6	436.7	49.2	478.9	25.5
	7	479.4	17.1	486.1	21.7
	8	505.7	32.2	528.9	24.6
	9	530.9	21.7	537.0	64.6
	10	612.0	49.5	643.3	30.6
	11	686.7	44.2	689.1	23.4
	12	715.6	215.3	765.0	271.7
	13	930.0	58.6	1002.3	66.4
	14	1043.3	66.0	1057.7	44.1

Table S2. Frequencies (f_{TOj}, f_{LOj}) and dampings (γ_{TOj}, γ_{LOj}) obtained from fitting polarized reflectivity spectra (**E**||**b**) in THz-IR range using Eqs. (1) and (2) with ε_∞=2.6.

Temperature	Number	f_{TOj} (cm^{-1})	γ_{TOj} (cm^{-1})	f_{LOj} (cm^{-1})	γ_{LOj} (cm^{-1})
**20 K**	1	85.7	17.7	89.1	17.5
	2	107.4	13.0	115.2	5.7
	3	115.4	5.4	125.4	12.7
	4	129.2	4.1	129.5	4.3
	5	137.9	3.8	139.7	2.9
	6	158.1	3.5	159.7	3.9
	7	178.8	5.3	180.3	5.0
	8	194.5	6.1	196.1	5.5
	9	219.2	8.5	219.6	5.8
	10	222.0	5.8	223.0	7.5
	11	231.6	9.7	231.9	10.3
	12	247.6	8.3	248.0	9.1
	13	253.7	5.4	255.5	3.4
	14	280.1	6.6	280.1	5.4
	15	284.9	7.2	285.5	6.3
	16	307.2	102.9	334.3	104.7
	17	349.0	11.6	354.6	7.1
	18	359.6	9.8	365.0	9.3
	19	365.4	10.6	374.6	10.7
	20	377.8	8.9	380.9	12.2
	21	381.7	15.5	397.8	12.8
	22	399.9	15.5	403.8	4.3
	23	442.1	55.6	466.8	56.6
	24	472.2	12.4	473.4	13.5
	25	484.3	15.0	492.4	8.8
	26	500.0	8.7	502.0	7.5
	27	504.8	11.7	524.6	10.0
	28	532.5	12.7	535.6	15.3
	29	548.4	11.7	550.6	13.2
	30	560.2	21.0	571.0	13.5
	31	580.8	22.4	597.0	14.2
	32	597.3	9.4	612.7	8.0
	33	612.8	7.8	638.5	5.5

**200 K**	1	57.8	46.7	97.0	42.0
	2	120.3	10.7	121.0	12.6
	3	127.3	10.3	131.6	8.9
	4	152.6	6.6	152.8	7.2
	5	161.7	10.8	163.4	12.8
	6	168.8	9.4	169.0	10.4
	7	180.1	13.2	181.9	11.2
	8	200.0	16.3	200.1	15.3
	9	221.5	5.5	222.0	5.0
	10	229.2	23.9	230.2	21.7
	11	238.9	7.7	239.0	7.7
	12	252.5	8.5	254.2	6.7
	13	263.0	10.3	263.0	9.4
	14	277.3	7.3	277.3	6.8
	15	285.8	8.0	285.9	7.4
	16	304.4	89.1	304.4	20.1
	17	306.9	20.6	334.3	72.0
	18	337.7	9.1	338.0	10.0
	19	348.3	17.2	354.0	17.8
	20	359.0	19.2	364.7	10.9
	21	365.2	10.9	373.0	13.9
	22	375.8	12.2	380.8	13.7
	23	380.8	18.3	399.3	10.4
	24	440.6	62.3	466.8	69.1
	25	470.4	18.4	472.3	19.0
	26	480.7	16.6	487.7	15.5
	27	502.2	25.4	512.4	6.4
	28	512.5	6.7	527.8	19.3
	29	535.4	14.9	535.9	21.6
	30	554.7	21.3	556.6	14.9
	31	558.1	19.5	570.8	25.2
	32	586.3	22.2	610.1	10.1
	33	610.3	10.3	638.3	11.0
**300 K**	1	87.8	39.5	119.9	28.7
	2	167.2	25.1	174.1	31.6
	3	174.4	9.3	175.3	10.5
	4	221.7	6.9	222.6	7.3
	5	249.7	17.1	252.4	17.7
	6	271.0	27.6	271.0	31.4
	7	353.8	21.9	394.6	14.6
	8	477.0	45.4	493.4	27.1
	9	507.8	22.3	522.8	19.3
	10	535.0	57.9	560.9	82.2
	11	593.2	27.0	642.1	24.0
	12	913.2	79.7	1008.7	37.6
	13	1010.1	34.9	1057.7	18.1

Table S3. Frequencies (f_{TOj}, f_{LOj}) and dampings (γ_{TOj}, γ_{LOj}) obtained from fitting polarized reflectivity spectra (**E**||**c**) in THz-IR range using Eqs. (1) and (2) with ε_∞=2.7.

Temperature	Number	f_{TOj} (cm^{-1})	γ_{TOj} (cm^{-1})	f_{LOj} (cm^{-1})	γ_{LOj} (cm^{-1})
**20 K**	1	3.1	49.4	4.3	49.8
	2	94.8	1.0	98.3	1.4
	3	110.1	48.3	112.0	30.2
	4	112.0	8.0	114.7	8.8
	5	129.7	31.7	139.4	43.3
	6	149.6	6.9	150.6	8.3
	7	175.6	29.3	182.7	41.0
	8	189.8	10.5	189.8	12.7
	9	201.9	4.4	205.1	6.2
	10	215.8	5.4	220.3	11.0
	11	226.3	10.7	238.1	8.2
	12	253.8	7.5	261.1	8.8
	13	262.3	4.4	264.6	4.8
	14	277.3	14.7	277.7	22.4
	15	291.1	7.6	291.9	8.0
	16	305.5	6.7	306.4	7.5
	17	325.0	21.5	337.5	18.1
	18	354.6	29.6	363.1	38.2
	19	370.4	6.6	371.7	8.1
	20	382.3	28.0	387.1	45.3
	21	398.8	10.1	405.0	8.6
	22	406.1	8.4	414.1	17.1
	23	425.6	5.7	428.9	11.4
	24	442.0	11.4	442.4	8.8
	25	453.3	11.3	453.3	13.1
	26	476.1	31.7	504.4	30.2
	27	508.0	13.5	517.4	12.7
	28	532.5	16.5	552.6	13.9
	29	559.3	11.6	561.0	9.0
	30	563.0	13.3	616.0	9.2

**200 K**	1	10.8	45.9	13.4	64.9
	2	94.9	5.0	98.6	2.1
	3	101.8	48.3	112.0	25.4
	4	112.0	18.5	116.9	25.1
	5	134.9	27.6	146.7	42.9
	6	151.5	16.8	152.7	23.7
	7	185.1	30.3	190.9	40.8
	8	197.3	14.0	197.6	17.4
	9	205.2	9.0	209.6	22.1
	10	222.5	20.7	233.5	13.6
	11	252.2	39.6	262.8	23.3
	12	277.1	17.2	277.7	23.2
	13	291.3	11.9	291.7	8.9
	14	303.6	35.8	316.8	43.4
	15	354.6	40.5	362.8	40.8
	16	371.7	9.9	373.1	9.7
	17	382.3	29.1	387.1	45.9
	18	400.5	19.5	406.0	9.1
	19	406.0	7.9	413.9	22.9
	20	423.0	9.6	429.1	12.9
	21	436.8	11.8	436.8	10.5
	22	452.9	11.9	453.3	13.1
	23	471.2	29.2	497.3	30.8
	24	506.6	19.8	514.8	13.8
	25	527.6	23.5	553.4	20.0
	26	559.8	18.4	617.7	11.5
**300 K**	1	10.1	31.5	16.0	74.7
	2	94.3	4.7	97.3	3.2
	3	102.4	108.3	151.8	150.6
	4	203.6	14.6	224.8	21.3
	5	232.4	27.2	240.0	22.3
	6	267.0	36.1	268.0	49.1
	7	353.7	9.3	353.9	10.2
	8	387.6	13.9	388.8	13.8
	9	406.6	19.5	410.8	10.4
	10	414.7	17.4	428.9	18.0
	11	469.8	36.5	508.1	21.9
	12	525.2	26.7	547.6	21.1
	13	549.5	9.8	550.5	10.1
	14	556.0	20.6	621.6	13.3

Table S4. Frequencies (f_{TOj}, f_{LOj}) and dampings (γ_{TOj}, γ_{LOj}) obtained from fitting polarized spectra (**E**||**c**) in LF-THz-IR range using Eqs. (1) and (2) with ε_∞=2.7.

Temperature	Number	f_{TOj} (cm^{-1})	γ_{TOj} (cm^{-1})	f_{LOj} (cm^{-1})	γ_{LOj} (cm^{-1})
**20 K**	1	3.1	49.4	4.3	49.8
	2	94.8	1.0	98.3	1.4
**50 K**	1	3.9	50.0	5.5	50.9
	2	94.8	1.0	98.3	1.4
**75 K**	1	4.0	51.0	5.8	53.8
	2	94.8	1.0	98.3	1.4
**100 K**	1	4.0	51.0	6.0	56.8
	2	94.8	1.7	98.3	1.9
**130 K**	1	2.0	51.0	2.7	51.1
	2	11.9	51.1	13.5	62.0
	3	94.8	2.7	98.8	1.9
**160 K**	1	2.0	51.0	2.6	51.1
	2	11.5	49.2	13.5	65.0
	3	94.8	3.4	98.6	2.1
**200 K**	1	2.0	51.0	2.4	51.1
	2	10.8	45.9	13.4	64.9
	3	94.9	5.0	98.6	2.1
**240 K**	1	2.0	51.0	2.4	51.1
	2	10.5	38.9	14.7	64.2
	3	94.9	6.7	98.6	2.8
**270 K**	1	2.0	51.0	2.3	51.1
	2	10.2	37.2	16.0	77.1
	3	94.3	4.7	97.3	3.2
**300 K**	1	2.0	51.0	2.4	51.1
	2	10.1	31.5	16.0	74.7
	3	94.3	4.7	97.3	3.2
